# Identification of a transcriptome profile associated with improvement of organ function in septic shock patients after early supportive therapy

**DOI:** 10.1186/s13054-018-2242-3

**Published:** 2018-11-21

**Authors:** Matteo Barcella, Bernardo Bollen Pinto, Daniele Braga, Francesca D’Avila, Federico Tagliaferri, Marie-Angelique Cazalis, Guillaume Monneret, Antoine Herpain, Karim Bendjelid, Cristina Barlassina

**Affiliations:** 1 Dipartimento di Scienze della Salute, Università degli Studi di Milano, Via Rudini 8, 20142 Milan, Italy; 2 Fondazione Filarete, Viale Ortles 22/4, 20139 Milan, Italy; 3 Department of Anaesthesia, Pharmacology and Intensive Care, Geneva University Hospitals, Rue Gabrielle-Perret-Gentil 4, Geneva, 1205 Switzerland; 4 Laboratoire Commun de Recherche HCL-bioMérieux, Hôpital Edouard Herriot, 376 Chemin de l’Orme, 6928 Marcy-l’Etoile Lyon, France; 5 Hospices Civils de Lyon, Hôpital Edouard Herriot, Laboratoire d’Immunologie, 5 Place d’Arsonval, 69437 Lyon cedex 03, France; 6 Department of Intensive Care, Hospital Erasme, Hospital, Université Libre de Bruxelles, Route de Lennik 808, Brussels, 1070 Belgium

**Keywords:** Critical illness, SOFA score, Gene expression, RNA-Seq, Septic shock

## Abstract

**Background:**

Septic shock is the most severe complication of sepsis and this syndrome is associated with high mortality. Treatment of septic shock remains largely supportive of hemodynamics and tissue perfusion. Early changes in organ function assessed by the Sequential Organ Function Assessment (SOFA) score are highly predictive of the outcome. However, the individual patient’s response to supportive therapy is very heterogeneous, and the mechanisms underlying this variable response remain elusive. The aim of the study was to investigate the transcriptome of whole blood in septic shock patients with different responses to early supportive hemodynamic therapy assessed by changes in SOFA scores within the first 48 h from intensive care unit (ICU) admission.

**Methods:**

We performed whole blood RNA sequencing in 31 patients: 17 classified as responders (R) and 14 as non-responders (NR). Gene expression was investigated at ICU admission (time point 1, or T1), comparing R with NR [padj < 0.01; Benjamini–Hochberg (BH)] and over time from T1 to T2 (48 h later) in R and NR independently (paired analysis, padj < 0.01; BH). Then the differences in gene expression trends over time were evaluated (Mann–Whitney, *P* <0.01). To identify enriched biological processes, we performed an over-representation analysis based on a right-sided hypergeometric test with Bonferroni step-down as multiple testing correction (padj < 0.05).

**Results:**

At ICU admission, we did not identify differentially expressed genes (DEGs) between the two groups. In the transition from T1 to T2, the activation of genes involved in T cell–mediated immunity, granulocyte and natural killer (NK) cell functions, and pathogen lipid clearance was noted in the R group. Genes involved in acute inflammation were downregulated in both groups.

**Conclusions:**

Within the limits of a small sample size, our results could suggest that early activation of genes of the adaptive immune response is associated with an improvement in organ function.

**Electronic supplementary material:**

The online version of this article (10.1186/s13054-018-2242-3) contains supplementary material, which is available to authorized users.

## Introduction

Sepsis is a life-threatening organ dysfunction caused by a dysregulated immune response to infection [1], and septic shock is its most severe form and is associated with an increased risk of mortality [2]. In addition to antibiotics or source control using surgery (or both), treatment of septic shock remains largely supportive of hemodynamics and tissue perfusion with fluids and vasopressors, which are associated with harmful side effects and can have a direct impact on immunity, organ function, and mortality [3, 4]. Early changes in organ function during the first days of an intensive care unit (ICU) stay as assessed by the Sequential Organ Function Assessment (SOFA) score are highly predictive of final outcome [5]. However, the response to supportive therapy is highly heterogeneous across patients, and the mechanisms underlying such variability remain elusive. In this context, it is not surprising that applying a “one-size fits all” protocol of fluids and vasopressors in early septic shock did not result in an amelioration of the outcome in all patients [6].

The characterization of patients at the molecular level is a promising approach to identify more specific targets for new therapeutic interventions in selected groups of patients [7]. Transcriptome profiling is a powerful tool to investigate and characterize the molecular mechanisms underlying specific physiological or pathological conditions.

In patients with septic shock, major transcriptomic changes occur within the first 48 h of shock development [8] and are associated with mortality in the ICU [9]. However, the reasons for this association remain largely unexplored. The authors hypothesized that this could be due to different trajectories of organ (dys)function in response to standard therapy.

We performed a whole blood RNA sequencing (RNA-Seq) analysis in septic shock patients with different responses to early supportive hemodynamic therapy as assessed by changes in SOFA scores within the first 48 h from ICU admission. The aim of the study was to identify gene expression profiles that distinguish patients as early improving (responders) or not (non-responders) on the basis of their SOFA score following initial therapy. Some of the results of this study have been previously reported in the form of an abstract [10].

## Methods

A detailed description of the methods is provided in Additional file 1.

### Study design and participants

This study is part of the multicenter prospective observational trial ShockOmics (ClinicalTrials.gov Identifier: NCT02141607) [11]. The ICUs involved are located in the Hopitaux Universitaires de Genève, Universite de Geneve (Geneva, Switzerland) and Hospital Erasme, Universite Libre de Bruxelles (Brussels, Belgium). The ethics committees of the two participating institutions approved the clinical protocol. Informed consent was obtained from the patients or their representatives. As previously described [11], we included consecutive adult (>18 years old) patients admitted for septic shock in the ICUs of two university tertiary hospital centers with an admission SOFA score at least 6 and an arterial lactate at least 2 mmol/L. Septic shock was defined in accordance with the current recommendations and international guidelines at that time [12]. Exclusion criteria included expected death within 24 h of ICU admission; more than 4 units of red blood cells or more than 1 fresh frozen plasma transfused; active hematological malignancy; metastatic cancer; chronic immunosuppression; pre-existing end-stage renal disease requiring renal replacement therapy; recent cardiac surgery; Child-Pugh C cirrhosis; and terminal illness.

We used SOFA score changes between time point 1 (T1), which was within 16 h of ICU admission, and time point 2 (T2), which was 48 h after study enrolment as an index of response to early supportive treatment to stratify subjects as responders (Rs) or non-responders (NRs) as previously reported [13]. A subject was classified as NR if the difference in SOFA score between T1 and T2 was less than 5 and the SOFA score at T2 was greater than 8. Patients who did not meet these criteria were classified as R.

### Blood collection and RNA extraction

Peripheral blood was collected in EDTA tubes at T1 and T2 and stored at −20 °C after adding 400 μL of 2X Denaturing solution (Ambion, Austin, TX, USA). Total RNA was extracted from 800 μL of blood using the MirVana Paris Kit and treated with Turbo DNA-free Kit (Ambion). RNA quality was assessed on an Agilent Bioanalyzer using the RNA 6000 Nano Kit (Agilent, Santa Clara, CA, USA), and samples were considered suitable for processing if the RNA integrity number was greater than 7.5.

### Library preparation

Sequencing libraries were prepared using TruSeq Stranded Total RNA obtained from the Ribo-Zero Globin Kit (Illumina, San Diego, CA, USA) using 800 ng of total RNA. Final libraries were validated with the Agilent DNA1000 kit and sequenced on a HiSeq2500 platform, producing 50 × 2-base pair paired-end reads.

### Sequencing data analysis

High-quality paired-end reads were aligned to the human reference genome (GRCh38) using STAR (version 2.5.2b) [14], and only uniquely mapping reads were used. Reads were assigned to genes with featureCounts (version 1.5.1) [15] using the gencode (version 25) primary assembly gene transfer file (GTF) as a reference annotation file for genomic feature boundaries.

### Differential expression analysis

Data preprocessing, exploratory data analysis, and analysis of differential gene expression were performed using DESeq2 package [16] built-in functions. We studied gene expression differences between R and NR at T1 and gene expression changes over time. These data were compared between T2 to T1 in R and NR groups, separately, using paired analysis. For both analyses, genes with padj < 0.01—Benjamini–Hochberg multiple test correction (FDR)—were considered differentially expressed (DEGs).

After selecting all the genes identified as DEGs in R or NR over time, we applied a Mann–Whitney test to compare T2 versus T1 fold changes between R and NR groups to identify DEGs with significant different trends between R and NR. The significance threshold was set to a *P* value of less than 0.01.

### Biological processes analysis

We used ClueGO version 2.3.4 [17] to identify enriched biological processes starting from the lists of DEGs identified in the over-time analysis in R and NR and the DEGs common to the two groups. The lists were used as input for the analysis after filtering for padj < 0.001. We performed an over-representation analysis (ORA) based on a right-sided hypergeometric test that uses Bonferroni step down as multiple testing correction. Enriched terms were grouped using kappa statistics, and only enriched clusters with a *P* value of less than 0.05 were considered statistically significant.

### Validation in an external dataset

To validate the DEGs identified in the transition from ICU admission to 48 h later, we searched in Gene Expression Omnibus (GEO) and Array Express repositories for gene expression datasets of septic shock patients studied at multiple time points. The microarray expression dataset GSE57065 included 28 septic patients studied at the onset of shock (that is, within 30 min after the beginning of vasoactive treatment, H0) and 24 and 48 h after (H24 and H48) [8]. We stratified patients as Rs or NRs using SOFA score changes between H0 and H48 as described in the “Study design and participants” section. Patients missing the H48 blood sample were excluded from the validation that was performed on 17 Rs and 9 NRs. We compared gene expression between H0 and H48 in the R and NR groups, separately. We established the analysis modifying the GEO2R script to take into account paired data [18]. Genes with padj < 0.01—Benjamini–Hochberg multiple test correction (FDR)—and log2FC > |0.5| were considered DEGs.

## Results

### Clinical variables

The study included 31 patients with septic shock enrolled between November 2014 and March 2016. In total, 17 patients were classified as R and 14 were classified as NR. The clinical characteristics of Rs and NRs at T1 and T2 are summarized in Table 1.

**Table 1:** Clinical variables

	Responders (*n* = 17)	Non-responders (*n* = 14)	*P* value
Demographic parameters			
Age, years	62.8 (18.4)	68.6 (20.7)	0.42
Sex, female	5 (29%)	5 (36%)	0.71
Body mass index, kg/m^2^	27.0 (4.6)	26.9 (6.4)	0.98
Source of infection			
Abdominal	5 (29%)	5 (36%)	0.22
Respiratory	4 (24%)	7 (50%)	
Urinary tract	6 (35%)	1 (7%)	
Other	2 (12%)	1 (7%)	
Clinical parameters			
Length of stay in ICU, days	6.2 (4.9)	11.9 (6.3)	0.01
Length of stay in hospital, days	21.0 (18.6)	28.7 (17.9)	0.31
Death in ICU	2 (12%)	4 (29%)	0.24
Death in hospital	2 (12%)	5 (36%)	0.11
APACHE II	23.1 (6.8)	25.1 (7.2)	0.42
SOFA T1	11.6 (3.0)	12.4 (2.4)	0.39
SOFA T2	6.1 (1.6)	11.4 (2.6)	*P* <0.001
Temperature, °C, T1	37.2 (1.2)	37.8 (1.2)	0.17
Temperature, °C, T2	37.8 (0.8)	37.2 (0.8)	0.02
Lactate, mmol/L, T1	4.0 (1.8)	5.3 (2.8)	0.17
Lactate, mmol/L, T2	1.5 (0.8)	2.1 (0.9)	0.10
SvcO_2_, %, T1	75.0 (5.57)	74.36 (7.72)	0.82
SvcO_2_, %, T2	68.5 (6.84)	69.33 (9.38)	0.84
Heart rate, bpm, T1	105.8 (28.8)	109.2 (17.7)	0.69
Heart rate, bpm, T2	89.2 (23.2)	95.4 (21.8)	0.46
Mean arterial pressure, mm Hg, T1	60.1 (4.7)	57.4 (7.3)	0.24
Mean arterial pressure, mm Hg, T2	67.5 (9.9)	66.0 (7.0)	0.62
Hydrocortisone T1	0 (0%)	0 (0%)	–
Hydrocortisone T2	1 (6%)	0 (0%)	0.36
Vasopressor treatment T1	17 (100%)	14 (100%)	0.36
Vasopressor treatment T2	3 (18%)	14 (100%)	*P* <0.001
Norepinehrine dose, μg/kg per min, T1	0.26 (0.19)	0.49 (0.38)	0.06
Norepinehrine dose, μg/kg per min, T2	0.09 (0.04)	0.24 (0.33)	0.12
Fluid balance, mL, T1	2229 (2468)	3498 (1943)	0.12
Fluid balance, mL, T2	135 (1203)	1566 (2062)	0.03
Urinary output, mL, T1	1674 (745)	1194 (750)	0.09
Urinary output, mL, T2	2273 (849)	2040 (1691)	0.65
Creatinine, mg/dL, T1	1.73 (1.17)	1.65 (0.83)	0.83
Creatinine, mg/dL, T2	1.20 (1.01)	1.46 (0.95)	0.46
Renal replacement therapy T1	1 (6%)	0 (0%)	0.36
Renal replacement therapy T2	0 (0%)	0 (0%)	–
Mechanical ventilation T1	13 (76%)	13 (93%)	0.22
Mechanical ventilation T2	9 (53%)	11 (79%)	0.14
paO_2_/FiO_2_, mm Hg, T1	203.9 (110.5)	205.7 (147.4)	0.97
paO_2_/FiO_2_, mm Hg, T2	271.0 (102.3)	219.4 (48.6)	0.08
Total leukocyte count, 10^3^/mm^3^, T1	15.92 (15.37)	15.35 (9.27)	0.90
Total leukocyte count, 10^3^/mm^3^, T2	14.08 (9.05)	16.76 (8.53)	0.42
Lymphocyte count, 10^3^/mm^3^, T1	0.85 (0.53)	1.00 (0.93)	0.60
Lymphocyte count, 10^3^/mm^3^, T2	0.90 (0.53)	0.72 (0.45)	0.34
C-reactive protein, mg/L, T1	245.2 (127.1)	217.0 (156.0)	0.59
C-reactive protein, mg/L, T2	224.2 (110.2)	248.1 (127.1)	0.59
Fibrinogen, mg/L, T1	5.34 (2.30)	4.60 (1.10)	0.27
Fibrinogen, mg/L, T2	5.98 (2.28)	5.81 (2.06)	0.85

Abbreviations: *APACHE II* Acute Physiology and Chronic Health Evaluation II, *ICU* intensive care unit, *paO*_*2*_*/FiO*_*2*_ partial pressure of oxygen/fraction of inspired oxygen, *SOFA* Sequential Organ Function Assessment, *SvcO*_*2*_ central venous oxygen saturation

Clinical characteristics of the patients divided in responders and non-responders at the two time points. Data are presented as mean (standard deviation) or frequency (percentage)

At T1, no significant differences were noted between the two groups in terms of variables describing the severity of organ dysfunction, whereas, consistent with our definition of R and NR, the SOFA score at T2 was increased in NRs (*P* <0.001). No significant differences were noted between the two groups regarding the source of infection, circulating markers of inflammation, or leukocyte and lymphocyte counts. The length of the ICU stay was longer in NR patients (*P* <0.05). More patients died in the ICU in the NR group (4 out of 14 in NRs versus 2 out of 17 in Rs); however, this difference was not statistically significant.

### Sequencing experiment

Total RNA libraries were sequenced in several batches, producing 31.07 M ± 6.92 M and 29.33 M ± 7.26 M raw read pairs on average for Rs and NRs, respectively. Ribosomal depletion was effective for all samples; the rRNA rate on mapped data was negligible in both groups (0.80 ± 0.95% and 0.78 ± 0.82% for Rs and NRs, respectively). The percentages of reads mapping to exons (86.09 ± 5.83% exonic rate) and DNase efficiency (2.75 ± 2.02% intergenic rate) were satisfactory in all samples. We obtained on average 13.67 ± 3.77 and 13.41 ± 3.69 million of uniquely and unambiguously mapped fragments for Rs and NRs, respectively (Additional file 2: Table S1). Globin reduction was successful with average percentages of counts mapping on globin genes of approximately 0.13% and 0.14% for NRs and Rs, respectively. Moreover, depletion efficiency was not statistically different for any of the genes coding for globins (Additional file 3: Table S7). Although we cannot test whether globin reduction influences sequencing efficiency of non-globin genes, this would equally affect both NR and R samples and thus is likely to bias toward the null.

### Gene expression analysis at ICU admission

**Fig. 1:**
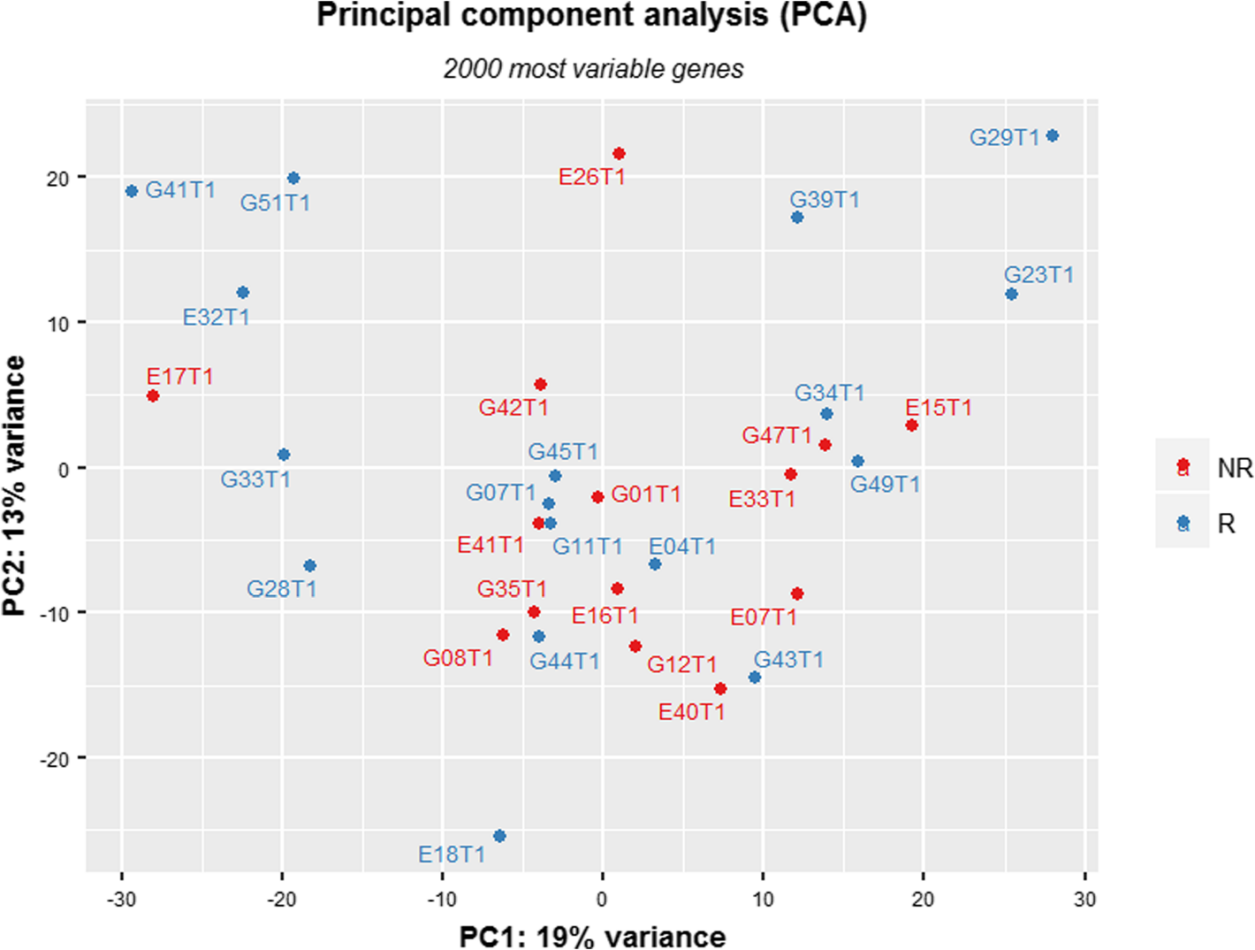
Principal component analysis of gene expression data in responders (Rs) and non-responders (NRs) at time point 1 (T1). The figure presents the two major principal components using the set of 2000 most variable genes (in expression) across all samples. Red describes NR patients whereas blue describes R patients. R and NR patients do not exhibit separation on the PC1 and PC2

We explored whether the transcriptome profile could distinguish Rs from NRs at ICU admission (T1). Principal component analysis was performed on the 2000 most variable genes across samples and did not reveal any separation between the R and NR groups (Fig. 1). Indeed, we could not identify any statistically significant DEG at T1 between Rs and NRs (padj < 0.01).

### Gene expression analysis over time

**Fig. 2:**
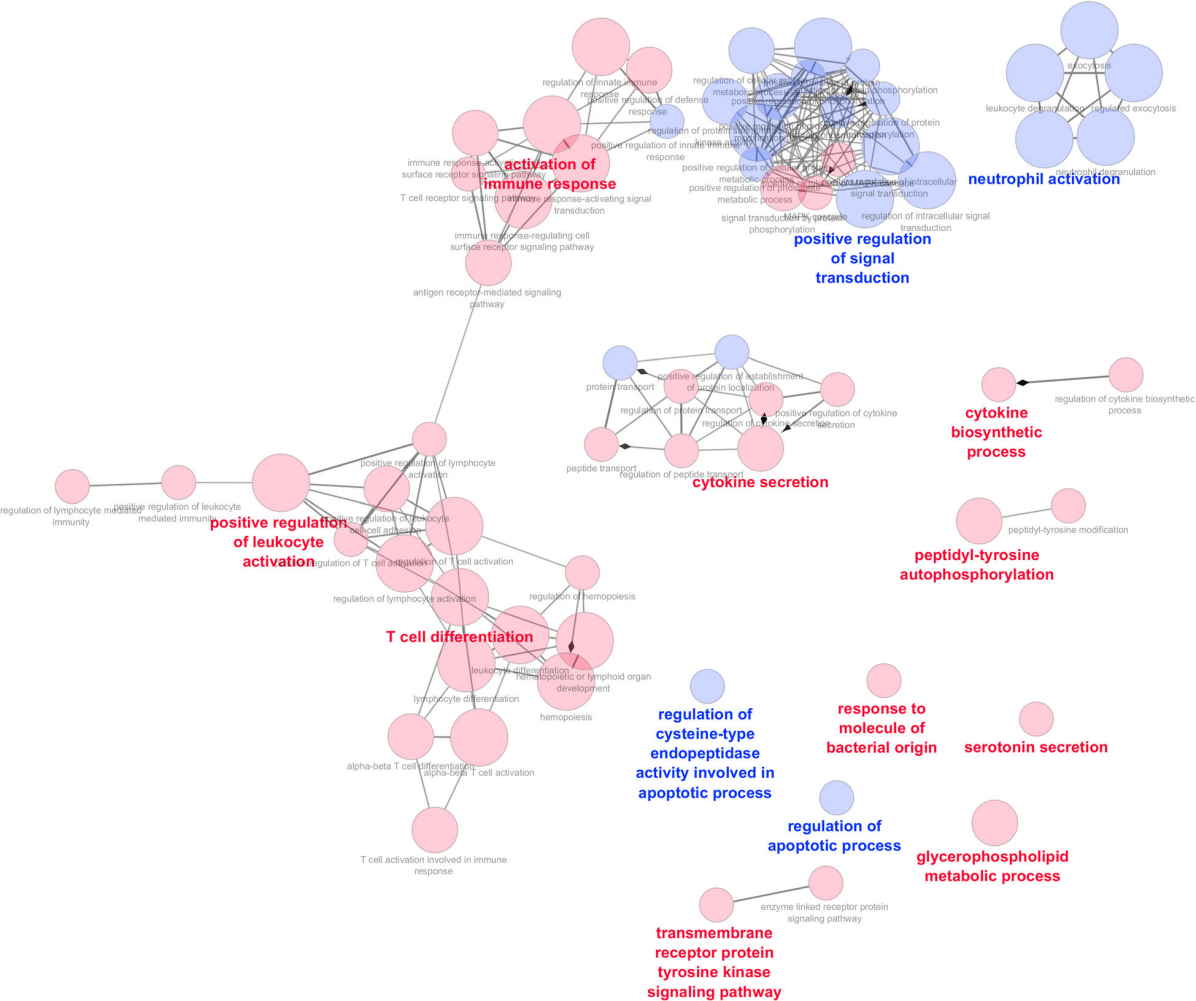
Network of enriched Gene Ontology (GO) term clusters in responder (R) patients. In this plot, each circle corresponds to an enriched GO term. GO terms enriched exclusively in R patients are noted in red, whereas those common to non-responder (NR) patients are noted in blue. The most significant enriched GO terms (leading GO terms) in each GO cluster are noted in bold

**Fig. 3:**
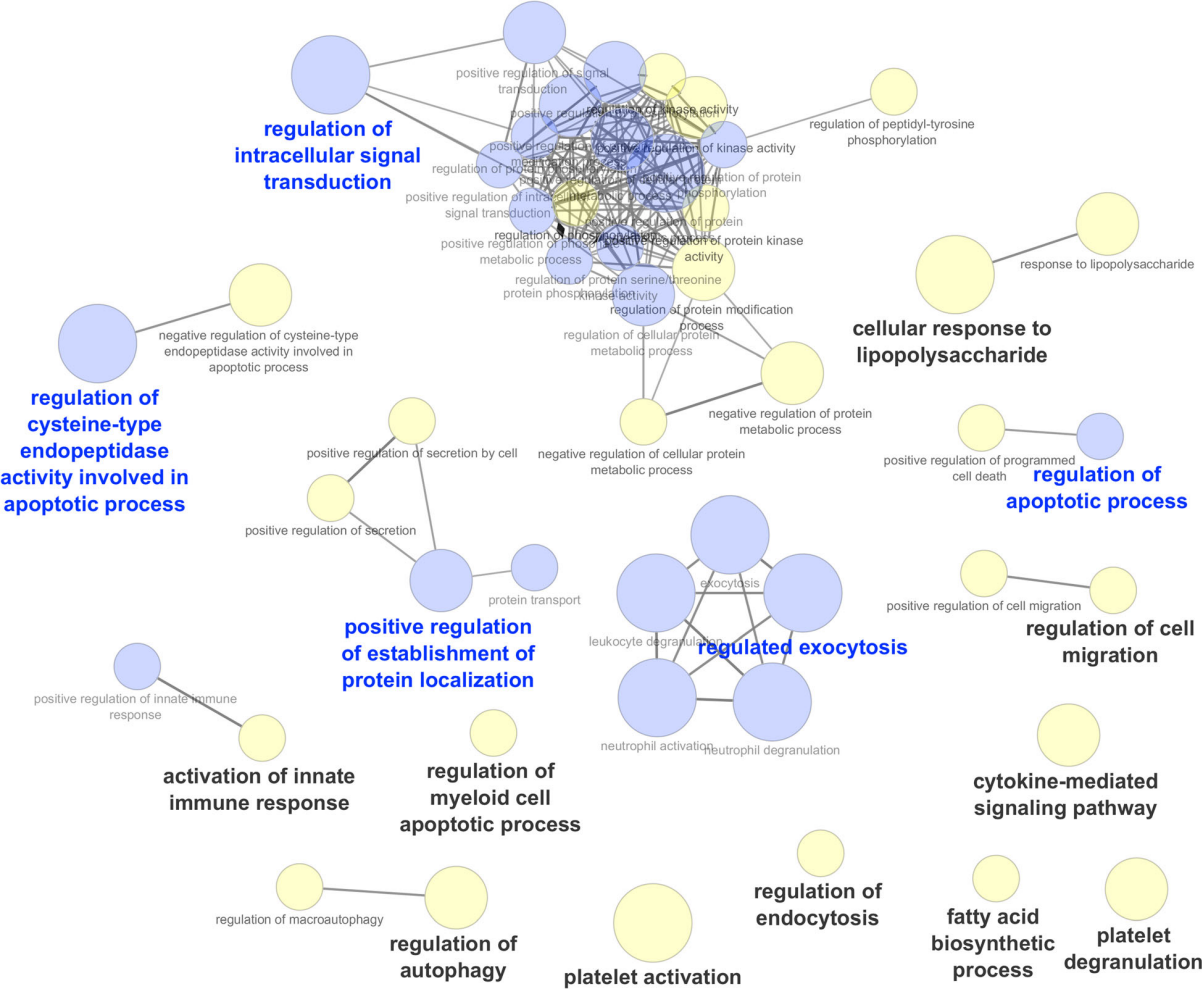
Network of enriched Gene Ontology (GO) term clusters in non-responder (NR) patients. In this plot, each circle corresponds to an enriched GO term. GO terms exclusively enriched in NR patients are noted in yellow, whereas those common to responder (R) patients are noted in blue. The most significant enriched GO terms (leading GO terms) in each GO cluster are noted in bold

We compared gene expression profiles between T2 and T1 in the R and NR groups independently. This analysis generated two lists of DEGs with 1979 DEGs (865 upregulated and 1114 downregulated genes) in Rs (Additional file 4: Table S2) and 1914 DEGs (711 upregulated and 1203 downregulated genes) in NRs (Additional file 5: Table S3). The two lists of DEGs were filtered at a padj < 10^−3^, yielding 1146 and 1120 genes in Rs and NRs, respectively, and were used as input for an ORA. In Rs, 14 clusters of significantly over-represented GO terms were identified with padj < 0.05 (Table 2; Additional file 6: Table S4). In NRs, the same analysis identified 15 clusters of over-represented GO terms (Table 3; Additional file 7: Table S5). A comparison between the GO networks identified in Rs and NRs was performed in Cytoscape, highlighting GO terms specifically enriched exclusively in Rs or NRs or enriched in both groups (Figs. 2 and 3). In Rs, we identified three robust GO clusters with highly interconnected GO terms (positive regulation of leukocyte activation, activation of immune response, and T-cell differentiation) (Fig. 2) that were not identified in NRs (Table 2), suggesting that the adaptive immune response is activated mostly in Rs at T2.

**Table 2:** Gene Ontology analysis in responder patients

GO groups	Group-adjusted *P* value	Leading GO ID	Leading GO term	Leading GO genes
Group 00	6.37E-5	GO:0001820	Serotonin secretion	6
Group 01	3.99E-5	GO:0006650	Glycerophospholipid metabolic process	44
Group 02	4.59E-5	GO:0042981	Regulation of apoptotic process	124
Group 03	7.67E-5	GO:0043281	Regulation of cysteine-type endopeptidase activity involved in apoptotic process	29
Group 04	8.10E-5	GO:0002237	Response to molecule of bacterial origin	40
Group 05	5.76E-5	GO:0007169	Transmembrane receptor protein tyrosine kinase signaling pathway	70
Group 06	6.17E-5	GO:0038083	Peptidyl-tyrosine autophosphorylation	14
Group 07	6.05E-5	GO:0042089	Cytokine biosynthetic process	20
Group 08, Group13	1.12E-7, 1.25E-16	GO:0002696	Positive regulation of leukocyte activation	48
Group 09	8.82E-20	GO:0042119	Neutrophil activation	92
Group10	6.79E-5	GO:0050663	Cytokine secretion	30
Group11	3.85E-12	GO:0002253	Activation of immune response	81
Group12	1.44E-9	GO:0009967	Positive regulation of signal transduction	149
Group13	1.25E-16	GO:0030217	T-cell differentiation	43

Gene Ontology (GO) analysis was performed on the most significant 1146 differentially expressed genes in responders between time point 1 (T1) and T2. Each line reports a significantly enriched GO group with its adjusted *P* value. The name of the most significant GO term in the GO group is reported together with the number of genes in the GO term

**Table 3:** Gene Ontology analysis in non-responder patients

GO groups	Group-adjusted *P* value	Leading GO ID	Leading GO term	Leading GO genes
Group 00	1.83E-5	GO:0019221	Cytokine-mediated signaling pathway	69
Group 01	1.14E-4	GO:0006633	Fatty acid biosynthetic process	20
Group 02	4.52E-5	GO:0030100	Regulation of endocytosis	28
Group 03	1.32E-6	GO:0030168	Platelet activation	28
Group 04	1.08E-4	GO:0033032	Regulation of myeloid cell apoptotic process	9
Group 05	7.87E-6	GO:0002576	Platelet degranulation	23
Group 06	8.84E-6	GO:0010506	Regulation of autophagy	39
Group 07	7.56E-5	GO:0002218	Activation of innate immune response	34
Group 08	1.18E-5	GO:0071222	Cellular response to lipopolysaccharide	29
Group 09	4.65E-5	GO:0030334	Regulation of cell migration	69
Group10	5.26E-5	GO:0042981	Regulation of apoptotic process	122
Group11	1.29E-6	GO:0043281	Regulation of cysteine-type endopeptidase activity involved in apoptotic process	33
Group12	1.78E-5	GO:1904951	Positive regulation of establishment of protein localization	56
Group13	5.59E-25	GO:0045055	Regulated exocytosis	122
Group14	3.21E-10	GO:1902531	Regulation of intracellular signal transduction	152

Gene Ontology (GO) analysis was performed on the most significant 1120 differentially expressed genes in non-responders between time point 1 (T1) and T2. Each line reports a significantly enriched GO group with its adjusted *P* value. The name of the most significant GO term in the GO group is reported together with the number of genes in the GO term

In NRs, we could not identify any GO cluster with highly interconnected GO terms with the exception of clusters of signal transduction and regulated exocytosis, which share many GO terms with Rs (Fig. 3). Among the GO clusters specifically enriched only in NRs, we highlighted the “Response to lipopolysaccharide” GO cluster, including CCL5, CXCL1, CXCL5, IRAK1, and NLRP3 genes that were exclusively downregulated in NRs. Analysis of the 752 DEGs common to Rs and NRs identified eight clusters of significantly enriched GO terms (Table 4) that are related to modulation of genes involved in the innate immune response and neutrophil function.

**Table 4:** Gene Ontology analysis on common differentially expressed genes between responder and non-responder patients

GO groups	Group-adjusted *P* value	GO ID	GO term	Number of genes	Leading GO term	Term-adjusted *P* value
Group 00	6.44E-05	GO:0050663	Cytokine secretion	22	x	1.11E-02
Group 01	7.17E-05	GO:0051248	Negative regulation of protein metabolic process	73	x	1.64E-02
Group 02	3.01E-06	GO:0002576	Platelet degranulation	19	x	4.18E-04
Group 03	6.65E-05	GO:0030168	Platelet activation	18	x	4.55E-02
Group 04	7.64E-05	GO:0002237	Response to molecule of bacterial origin	30		2.62E-02
Group 04	7.64E-05	GO:0071219	Cellular response to molecule of bacterial origin	23	x	5.42E-04
Group 05	5.59E-08	GO:0009967	Positive regulation of signal transduction	95	x	4.14E-04
Group 05	5.59E-08	GO:1902531	Regulation of intracellular signal transduction	107		2.07E-03
Group 06	1.39E-25	GO:0006887	Exocytosis	106		7.79E-24
Group 06	1.39E-25	GO:0042119	Neutrophil activation	78		7.11E-23
Group 06	1.39E-25	GO:0045055	Regulated exocytosis	102	x	5.45E-26
Group 06	1.39E-25	GO:0043299	Leukocyte degranulation	81		1.03E-22
Group 06	1.39E-25	GO:0043312	Neutrophil degranulation	77		1.32E-22
Group 07	1.64E-06	GO:0002253	Activation of immune response	54		3.68E-04
Group 07	1.64E-06	GO:0031349	Positive regulation of defense response	37		2.91E-03
Group 07	1.64E-06	GO:0002757	Immune response-activating signal transduction	47		5.24E-03
Group 07	1.64E-06	GO:0045088	Regulation of innate immune response	39	x	8.43E-05
Group 07	1.64E-06	GO:0045089	Positive regulation of innate immune response	31		3.38E-03
Group 07	1.64E-06	GO:0002218	Activation of innate immune response	28		4.86E-03

Gene Ontology (GO) analysis was performed on 752 differentially expressed genes identified between time point 1 (T1) and T2 in both responders and non-responders. Each line reports a significantly enriched GO term with its adjusted *P* value, the number of genes included and the GO group to which they belong

### Gene expression trend analysis

**Fig. 4:**
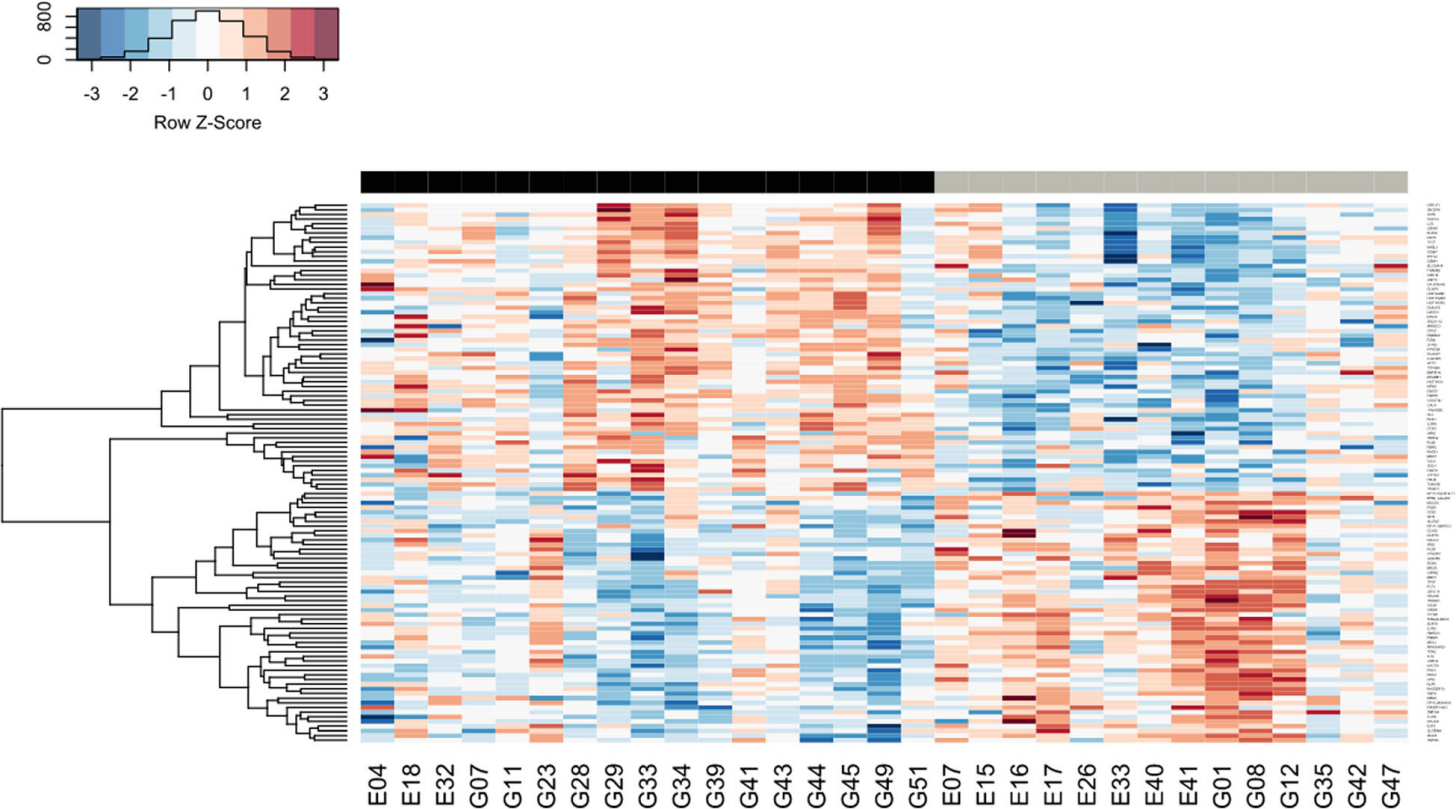
Genes with significantly different time point 1/time point 2 (T1/T2) expression trends between responder (R) and non-responder (NR) septic shock patients. Each column represents the gene expression profile of a patient. Black bar on the top indicates Rs whereas gray bar indicates NRs. The fold expression change is represented by a color scale gradient from blue (downregulation at T2) to red (upregulation at T2)

We identified 125 genes (*P* <0.01, Mann–Whitney) with significantly different trends between Rs and NRs in the transition from T1 to T2 (Fig. 4; Additional file 8: Table S6). Thirteen genes (CD247, FCER1A, ICOS, LCK, SH2D1A, CLC, IRAK3, VSIG4, GPR171, GPR18, HIST1H3A, ZNF784, and TIGIT) among 125 are involved in T-cell functions and support the results highlighted in the GO analysis that Rs modulate biological processes of the adaptive immune response.

We observed that, among the 125 genes, genes involved in granulocyte functions (IL5RA, FCER1A, CCR3, and PTGDR2) and genes involved in natural killer (NK) cell activity (GZMM, GZMH, and KLRG1) were significantly upregulated at T2 exclusively in Rs. We also identified PCSK9 and SORT1 genes, which are involved in the lipid pathway. PCSK9 is significantly downregulated in Rs at T2. In contrast, SORT1 is downregulated at T2 in both groups, but different fold changes are noted for each group. We also observed genes with a role in the maintenance of vascular tone and endothelial function, such as CPA3 (upregulated at T2 in Rs), ARG1 (downregulated at T2 in Rs), and AVPR1A (downregulated at T2 in NRs).

### Validation in an external dataset

Patients of the validation (GSE57065) and of the present study had similar clinical characteristics except for the SOFA score at T1 in Rs, which was higher in the present cohort compared with the validation one (11.58 ± 3 versus 9.29 ± 2.49, *P* = 0.026). Gene expression comparison between H0 and H48 in the validation cohort generated two lists of 590 DEGs (327 upregulated and 263 downregulated) in Rs and 996 DEGs (819 upregulated and 178 downregulated) in NRs. To assess concordance of gene ID and direction of regulation, we compared these two lists with the DEG lists obtained for the R and NR groups in our study. We identified 234 DEGs modulated in Rs and 245 DEGs modulated in NRs in both datasets (padj < 0.05 [BH] and logFC > |0.5|). The concordance (upregulation/downregulation) was high (231 out of 234 for Rs and 242 out of 245 for NRs).

## Discussion

We explored the transcriptome profile associated with early response to hemodynamic supportive therapy using whole blood of patients with septic shock to identify gene expression profiles that distinguish Rs from NRs over the first 48 h from ICU admission. This time window is crucial for critically ill patients given that those who exhibit improved organ function exhibit a better outcome [5, 19–21]. To quantify gene expression, we used RNA-Seq, which, compared with microarray, offers increased dynamic range of signal and reproducibility [22]. Moreover, RNA-Seq can interrogate the whole transcriptome and not only those genes for which the probes are designed on the microarray.

The comparison of the transcriptomic profile at ICU admission (T1) between Rs and NRs did not reveal any statistically significant difference. Previous studies with larger cohorts successfully identified a relation between gene expression profiles in leukocytes or whole blood cells at ICU admission and survival in sepsis and septic shock [23, 24]. Our negative result could be explained by the fact that our cohort is composed exclusively of septic shock patients highly homogeneous for disease severity, given that all patients exhibit high SOFA scores and are treated with vasopressors. Gene expression in the two groups likely reflects the homogeneity of the clinical phenotype. This result is consistent with that observed in a recent metabolomics study performed in a subgroup of these patients with identical metabolomic profiles at ICU admission [13].

Gene expression analysis over time was performed separately in Rs and NRs with a paired analysis leveraging the correlation between T1 and T2 and improving statistical power. The upregulation of neutrophil bacterial killing genes (CTSG, ELANE, DEFA4, and AZU1) and Toll-like receptor (TLR) genes involved in recognition of ssRNA (TLR7) and anti-inflammatory functions (TLR10) was detected at T2 in both Rs and NRs. In contrast, the following genes related to acute inflammation were downregulated: C-type lectin receptors (CLEC6A, CLEC4D, and CLEC5A), interferon receptors (IFNAR1 and IFNGR1), alarmins (S100A8 and S100A12), and metalloproteases involved in extracellular matrix remodeling (MMP8 and MMP9).

Interestingly, a comparison of the biological networks between Rs and NRs highlighted that R patients specifically modulate genes involved in T-cell activation but that NR patients do not, as observed in the trend analysis. In detail, ICOS and LCK, two important co-stimulatory molecules of T-cell activation [25, 26], and CD247, the major signal transduction element in the T-cell antigen receptor complex, were upregulated in Rs at T2. Modulation of these genes has been observed in relation to survival in previous articles [23, 27]. VSIG4, a strong inhibitory molecule of T-cell activation [28], was downregulated in Rs and upregulated in NRs at T2. The activation of adaptive immunity genes in Rs could suggest improved ability to eradicate infections [29] and reduce inflammation-induced organ injury. Consistent with this consideration, some genes with a relevant function in bacterial killing (CCL5, CXCL1, CXCL5, IRAK1, and NLRP3) were exclusively downregulated in NRs at T2.

The analysis of gene expression trends identified 125 genes differentiating the two groups of patients. However, a certain degree of heterogeneity of gene expression changes can be appreciated in each group (Fig. 4) that can likely be explained by the underlying causes of the shock condition and patient-specific factors [30, 31].

Among these genes, serin protease genes (GZMM, GZMH, and KLRG1) involved in NK cell activity and cell-mediated immune response [32] and genes expressed mainly in granulocytes (CLC, CCR3, PTGDR2, FCER1A, and IL5RA) were exclusively upregulated at T2 in R patients. Recent studies have reported the association between granulocytes and sepsis [33]. CLC (Charcot-Leyden crystal galectin) encodes a lysophospholipase that regulates the immune response through the recognition of cell surface glycans. CCR3, PTGDR2 (alias CRTH2), and FCER1A are associated with allergic processes and have been described in sepsis [34] and bacterial meningitis [35]. IL-5RA is the receptor of IL-5, which has been proposed as a viable therapy for sepsis and is required for attraction and activation of eosinophils by Th2 cells [36]. Among the genes that differentiate R and NR patients, IL-1R2 was exclusively downregulated in R. It encodes interleukin 1 receptor type 2, which acts as a decoy receptor for IL-1A, inhibiting its pro-inflammatory activity [37]. Recently, increased IL-1R2 expression has been noted in septic patients compared with controls [38], and IL-1R2 has been proposed as a new biomarker for sepsis diagnosis.

Trend analysis revealed that PCSK9 and SORT1, two genes involved in lipid clearance, are downregulated in Rs. PCSK9 inhibits the clearance of endogenous cholesterol lipids from plasma by decreasing the number of LDL receptors that are involved in the removal of bacterial lipids from the blood during infections [39, 40]. SORT1 is a critical regulator of PCSK9 activity, promoting its secretion from primary hepatocytes [41]. In Rs, its modulation combined with the downregulation of PCSK9 could promote increased clearance of endogenous lipids and bacteria. Indeed, in our patient cohort, lipidome alterations were found to play an important role in individual patient response to infection [13].

Hypotension [42] and impairment of endothelial function [43, 44] are key pathophysiological features of septic shock. Exclusively in R patients, CPA3 encoding a carboxypeptidase that degrades toxic peptides *in vivo* was upregulated at T2. ARG1 and AVPR1A were downregulated in Rs and NRs, respectively. ARG1 encodes arginase that affects nitric oxide generation [45], impacting the regulation of vascular tone and maintenance of endothelial function [46]. The vasopressin receptor AVPR1A is also expressed in platelets [47] and is responsible for the vasoconstrictor activity of vasopressin [47, 48]. Decreased expression of AVPR1A receptors and reduced levels of vasopressin have been described in experimental models of sepsis and human sepsis, respectively [47, 49].

Because the relatively small sample size of our study may be considered a limitation, we validated the DEGs identified and highlighted in the discussion, using the external dataset GSE57065. We validated genes involved in bacterial killing and anti-inflammatory functions (DEFA4, AZU1, ELANE, CTSG, and TLR10), C-type lectin receptor genes (CLEC4D and CLEC5A), the interferon receptor gene IFNAR1, the metalloprotease gene MMP8, genes involved in lipid clearance (PCSK9 and SORT1), and the alarmin gene S100A8.

Moreover, to further confirm our results, we focused on the GAinS study [23] that investigated gene expression in 265 septic patients at ICU admission and identified two sepsis signatures: SRS1 associated with increased mortality and SRS2. Interestingly, genes involved in T-cell function (CD247, SH2D1A, ICOS, TIGIT, LCK, and CLC), which were associated with improvement of organ function in R patients in our cohort, were also associated with survival in the GAinS study, suggesting that their early modulation is critical for a favorable outcome. Furthermore, genes involved in granulocyte function (FCER1A and CCR3), NK cell activity (GZMM, GZMH, and KLRG1), lipid pathways (PCSK9 and SORT1), and endothelial function (ARG1 and CPA3) exhibited the same direction of modulation in survivors and the R group.

We chose to categorize our cohort according to changes in organ function and not mortality. The association between ICU mortality and whole blood transcriptomics has been previously described [9, 23], and major changes have been observed within the first 48 h of shock development [8]. However, the reason for this association remains largely unexplored. We hypothesized that this could be due to different trajectories of organ (dys)function in response to standard therapy in the first days of ICU stay, which is a key determinant of outcome [5, 21, 50]. However, the mechanisms remain incompletely characterized. In a recent article in *Critical Care*, treatment effects of different interventions on delta-SOFA are “reliably and consistently associated with mortality”, and delta-SOFA has been recommended as an endpoint in randomized controlled trials [51]. Finally, as the prevalence of ICU mortality decreases, methodological issues regarding statistical power are relevant. Hence, studying “intermediate” phenotypes that are more common than mortality and yet closely related is justified [52].

## Conclusions

This study highlights a specific transcriptome profile associated with a positive response to supportive therapy. In short, this profile is characterized by the activation in R patients of genes involved in T cell–mediated immunity and of genes related to granulocyte and NK cell function and by the modulation of genes involved in pathogen lipid clearance, which contribute to a more efficient infection eradication and inflammation modulation.

## Additional files

Additional file 1: Supplementary methods. (DOCX 15 kb)

Additional file 2: **Table S1.** Metrics and basic statistics of sequencing data. This table reports the number of reads processed along the data analysis workflow. In particular, it presented the number of sequenced reads (raw reads), the number of quality filters passing reads (trimmed reads), and the number of reads that map to exons (column E). Columns F to I report the percentage of reads mapping to rRNA and the rate of mapping along genic or intergenic regions. (XLSX 16 kb)

Additional file 3: **Table S7.** Globin depletion (Mann–Whitney test). This table summarizes the percentage of reads mapping unambiguously to the reference genome (GRCh38) regarding all genes encoding for globins. For each gene, the following information is reported: mean percentage in responders (Rs) and non-responders (NRs), standard deviation in Rs and NRs, and *P* value (Mann–Whitney test) of the comparison test between Rs and NRs. (XLSX 17 kb)

Additional file 4: **Table S2.** Differentially expressed gene (DEG) T2vsT1 in responder (R) patients. This table describes the results of the differential expression analysis between T1 and T2 in Rs. In total, 1979 DEGs were identified with a padj < 0.01. For each gene, the following information is reported: Ensembl Gene Name, the mean level of expression in all samples (baseMean), log2FoldChange comparing T2 versus T1, level of statistical significance (padj), genomic coordinates (chromosome_name, start_position, end_position), description of gene function (description), official gene symbol (external_gene_name), type of gene (gene_biotype), and the normalized gene count values in each biological sample. Abbreviations: *T1* time point 1, *T2* time point 2. (XLSX 2787 kb)

Additional file 5: **Table S3.** Differentially expressed gene (DEG) T2vsT1 in non-responder (NR) patients. This table describes the results of the differential expression analysis between T1 and T2 in NRs. In total, 1914 DEGs were identified with a padj < 0.01. For each gene, the following information is reported: Ensembl Gene Name, the mean level of expression in all samples (baseMean), log2FoldChange comparing T2 versus T1, level of statistical significance (padj), genomic coordinates (chromosome_name, start_position, end_position), description of gene function (description), official gene symbol (external_gene_name), type of gene (gene_biotype), and the normalized gene count values in each biological sample. Abbreviations: *T1* time point 1, *T2* time point 2. (XLSX 2409 kb)

Additional file 6: **Table S4.** Gene Ontology (GO) enriched clusters obtained from differentially expressed gene (DEG) list – full table in responder (R) patients. This table is an extended and more detailed version of Table 2. All of the 66 significantly enriched GO terms are described, and we specify whether the GO term is found exclusively in Rs or in both Rs and non-responders (NRs) (Patient condition). (XLSX 17 kb)

Additional file 7: **Table S5.** Gene Ontology (GO) enriched clusters obtained from differentially expressed gene (DEG) list – full table in non-responder (NR) patients. This table is an extended and more detailed version of Table 3. All of the 48 significantly enriched GO terms are described, and we specify whether the GO term is found exclusively in NRs or in both responders (Rs) and NRs (Patient condition). (XLSX 16 kb)

Additional file 8: **Table S6.** Trend analysis (Mann–Whitney test) – full table. This table describes the 125 genes exhibiting different expression trends in responders (Rs) and non-responders (NRs) between time point 1 (T1) and T2. For each gene, the following information is reported: log2FoldChange comparing T2 versus T1 in R (log2FC.R) and NR (log2FC.NR), the level of statistical significance in R and NR (padj.R, padj.NR), the level of significance of the Mann–Whitney test (pval.wilcox), the mean level of expression in R (basemean R) and NR (baseMean NR), genomic coordinates (chromosome, start, end), description of gene function (description), and type of gene (gene_biotype). (XLSX 33 kb)

## Abbreviations

BH: Benjamini–Hochberg
DEG: Differentially expressed gene
FDR: False discovery rate
GEO: Gene expression omnibus
GO: Gene Ontology
H: Hour
ICU: Intensive care unit
NK: Natural killer
NR: Non-responder
ORA: Over-representation analysis
R: Responder
RNA-Seq: RNA sequencing
SOFA: Sequential organ function assessment
SRS: Sepsis response signature
T1: Time point 1
T2: Time point 2
TLR: Toll-like receptor
