# Supplementary material for: Identification of a transcriptome profile associated with improvement of organ function in septic shock patients after early supportive therapy
Source: Crit Care. 2018 Nov 21;22:312. doi: 10.1186/s13054-018-2242-3 (PMC6249814; doi:10.1186/s13054-018-2242-3)
Supplement: Supplementary file 1 — Supplementary methods. (DOCX 15 kb) [file 13054_2018_2242_MOESM1_ESM.docx]

**Supplementary Methods**

**Blood collection and RNA extraction**

Peripheral blood was collected for each patient in EDTA tubes at T1, in the acute phase of shock within 16 hrs from ICU admission, and at T2, 48 hrs from ICU admission. Immediately after collection, 400µl of whole blood were added to 400µl of 2X Denaturing solution (Ambion, Austin, TX, USA) for proper RNA conservation, in duplicate, gently mixed and stored at -20°C.

Total RNA was extracted from 800 µl of treated blood with MirVana Paris Kit (Ambion, Austin, TX, USA) and treated with Turbo DNA-free Kit (Ambion, Austin, TX, USA). RNA Quality was assessed on Agilent Bioanalyzer with the RNA 6000 Nano Kit (Agilent, Santa Clara, CA). RNA Integrity Number (RIN) was determined for every sample and all samples were considered suitable for processing if RIN > 7.5. RNA concentration was spectrophotometrically estimated using Nanoquant Infinite M200 instrument (Tecan, Austria).

**Library preparation**

Sequencing libraries were prepared with the TruSeq Stranded Total RNA with Ribo-Zero Globin Kit (Illumina, San Diego, CA) using 800 ng of total RNA as input. The kit uses oligo-attached magnetic beads to remove rRNA and globin mRNA from total RNA. The RNAs were then purified, fragmented at 94°C for 8 minutes and primed with random hexamers for cDNA synthesis. All cDNAs were indexed and amplified with 11 PCR cycles. Final libraries were validated and quantified with the DNA1000 kit on Agilent Bioanalyzer. Libraries were pooled and sequenced on a HiSeq2500 platform producing 50x2 bp paired end reads.

**Sequencing data analysis**

Raw sequencing data were quality checked using the FastQC tool (v0.11.5) (http://www.bioinformatics.babraham.ac.uk/projects/fastqc/). In order to ensure the highest mapping quality, we performed a gentle trimming using Trimmomatic (v0.35) setting the following parameters: MINLEN:40, AVGQUAL:25 and TRAILING:15. High quality paired-end reads were then aligned to the human reference genome (GRCh38) using STAR (v2.5.2b) with –outFilterMultimapNmax 1 in order to emit and use only uniquely mapping reads. Mapping files were checked for several metrics including coverage distribution across gene length, ribosomal rRNA depletion efficiency, DNA contamination and percentage of reads mapping to exons. Post alignment metrics were collected by using RNA-SeQC (v1.1.8.1). Samples that did not pass post-alignment quality metrics (high rRNA rates or high percentage of intergenic mapping), were subjected to de-novo library preparation and re-sequenced.

We then assigned reads to genes (gene counting) by using featurecounts (v1.5.1), a highly customizable software for assigning reads to genomic features like genes, exons, and promoters. We setup parameters in order to use only fragments with both ends mapped and used reads overlapping the exons with at least 5 nucleotides (--minOverlap 5). We used the gencode (v.25) primary assembly gene transfer file (GTF) as reference annotation file for genomic features boundaries.

The resulting file is a matrix of counts in which the rows represent genes (58096 genes according to gencode v.25) whereas the columns represent the samples.

**Exploratory Data Analysis**

In order to figure out samples variability we performed Principal Component Analysis (PCA) starting from regularized log transformed counts matrices created by rlog function implemented in DESeq2 package and using prcomp function to perform PCA calculations. We used PCA as tool to formulate hypothesis. PCA charts were produced by ggplot2 package.

**Differential expression analysis**

In order to identify those differences in gene expression modulation that distinguish R from NR, we setup a 2-steps analysis. In the first step, we explored if differences in gene expression exist between R and NR in the acute phase of septic shock at ICU admission (timepoint T1). We used as input the raw counts matrix of the samples at T1 (31 patients and 58906 genes). Data preprocessing, exploration data analysis and statistical differential analysis of gene expression were performed by DESeq2 package build-in functions. We performed R to NR at T1 by setting counts ~ condition design and using p.adj < 0.01 as cut-off threshold for Wald test results. Genes with p.adj < 0.01 were considered differentially expressed (DEGs).

In the second step, we studied gene expression changes over time, comparing T2 to T1, in R and NR separately, and focused on genes specifically modulated in R or NR. For this aim, we performed a T2 vs T1 paired analysis of differential expression (counts ~ patient + timepoint) in R and NR separately. Genes were considered significantly differently expressed if the p.adj (Benjamin Hochberg multiple test correction (FDR)) of the test (Wald Test) was < 0.01. DEGs lists for R and NR were used as input for biological processes (BP) over expression analysis (ORA) and a network analysis, as described in Biological Processes analysis section.

**Gene expression trends analysis**

In order to identify the genes whose trend of expression from T2 to T1 was significantly different in R compared to NR, we initially selected all the genes identified as DEGs in R or NR comparing T2 to T1. We then removed genes whose base mean of expression was lower than 10 both in NR and in R. On the remaining genes, we built a matrix of log_2_Fold Change (T2/T1) for each sample. We then applied a Mann Whitney test (non-parametric) between R and NR for each gene. We discarded genes with p value of Mann Whitney test > 0.01. The list of genes was then additionally filtered by removing genes with |LogFC| < 0.5 in both groups.

**Biological processes analysis**

We used ClueGO v2.3.4 to identify enriched biological processes, starting from the lists of DEGs identified in R and NR. The two lists of DEGs with an adjusted p value < 0.001 were used as input for the analysis. Parameters were set up in order to decrease redundancy by fusing related terms that share similar gene sets. GO Terms used for the analysis were all the GO terms in a level from 6 to 10 in the GO tree (GO_BiologicalProcess-EBI-QuickGO-GOA_24.08.2017_00h00). We performed an overrepresentation analysis (ORA) based on a right-sided hypergeometric test that uses Bonferroni as multiple testing correction. Enriched terms were grouped through a Kappa Statistics (kappa score threshold 0.4) in order to reduce the complexity of the results. Only enriched clusters with a p-value < 0.05 were considered statistically significant. An interaction plot showing the significant GO terms and GO clusters was produced in ClueGO and subsequently analyzed in Cytoscape v3.5.1 (2003_Shannon). A further list of 752 DEGs common between R and NR (p-value < 0.001 in at least one of the two groups) was used as input for an ORA of common genes as described above.
